# Describing and Sharing Molecular Visualizations Using the MolViewSpec Toolkit

**DOI:** 10.1002/cpz1.1099

**Published:** 2024-07

**Authors:** Sebastian Bittrich, Adam Midlik, Mihaly Varadi, Sameer Velankar, Stephen K. Burley, Jasmine Y. Young, David Sehnal, Brinda Vallat

**Affiliations:** 1 Research Collaboratory for Structural Bioinformatics Protein Data Bank, San Diego Supercomputer Center, University of California, San Diego, La Jolla, California; 2 Protein Data Bank in Europe, European Molecular Biology Laboratory, European Bioinformatics Institute, Hinxton, Cambridge, United Kingdom; 3 Research Collaboratory for Structural Bioinformatics Protein Data Bank and the Institute for Quantitative Biomedicine, Rutgers, The State University of New Jersey, Piscataway, New Jersey; 4 Cancer Institute of New Jersey, Rutgers, The State University of New Jersey, New Brunswick, New Jersey; 5 Department of Chemistry and Chemical Biology, Rutgers, The State University of New Jersey, Piscataway, New Jersey; 6 National Centre for Biomolecular Research, Faculty of Science, Masaryk University, Brno, Czech Republic; 7 These authors contributed equally to this work.

**Keywords:** 3D visualization, interoperability, macromolecular structure, mmCIF, Protein Data Bank

## Abstract

With the ever-expanding toolkit of molecular viewers, the ability to visualize macromolecular structures has never been more accessible. Yet, the idiosyncratic technical intricacies across tools and the integration complexities associated with handling structure annotation data present significant barriers to seamless interoperability and steep learning curves for many users. The necessity for reproducible data visualizations is at the forefront of the current challenges. Recently, we introduced MolViewSpec (homepage: https://molstar.org/mol-view-spec/, GitHub project: https://github.com/molstar/mol-view-spec), a specification approach that defines molecular visualizations, decoupling them from the varying implementation details of different molecular viewers. Through the protocols presented herein, we demonstrate how to use MolViewSpec and its 3D view–building Python library for creating sophisticated, customized 3D views covering all standard molecular visualizations. MolViewSpec supports representations like cartoon and ball-and-stick with coloring, labeling, and applying complex transformations such as superposition to any macromolecular structure file in mmCIF, BinaryCIF, and PDB formats. These examples showcase progress towards reusability and interoperability of molecular 3D visualization in an era when handling molecular structures at scale is a timely and pressing matter in structural bioinformatics as well as research and education across the life sciences. © 2024 The Authors. Current Protocols published by Wiley Periodicals LLC.

**Basic Protocol 1**: Creating a MolViewSpec view using the MolViewSpec Python package

**Basic Protocol 2**: Creating a MolViewSpec view with reference to MolViewSpec annotation files

**Basic Protocol 3**: Creating a MolViewSpec view with labels and other advanced features

**Support Protocol 1**: Computing rotation and translation vectors

**Support Protocol 2**: Creating a MolViewSpec annotation file

## INTRODUCTION

The Protein Data Bank (PDB) (Crystallography: Protein Data Bank, 1971; Velankar et al., 2021; wwPDB consortium, 2019) is the single global repository for experimentally determined structures of macromolecules. Established in 1971, the PDB contains more than 220,000 atomic-level structures of proteins, nucleic acids, and their complexes with other macromolecules and a diverse array of small chemicals. Open-access macromolecular structure data through the PDB has been fundamental to understanding biological processes and elucidating molecular interactions and functions. Furthermore, visualizing three-dimensional (3D) structures of macromolecules and their interactions with small molecules/ligands is vital for understanding reaction mechanisms and deriving insights into biological function at the atomic level.

The recent emergence of computational structure prediction methods has led to a dramatic increase in the availability of predicted structures (Danneskiold-Samsøe et al., 2023; Rossi Sebastiano et al., 2022; Varadi et al., 2022), offering unparalleled insights into biological mechanisms at a molecular level (Bordin et al., 2023; Mosalaganti et al., 2022). New-generation AI-powered tools like AlphaFold (Jumper et al., 2021), ESMFold (Lin et al., 2023), RoseTTAFold (Baek et al., 2021), and others employ advanced algorithms to provide a bounty of predicted protein structures (or computed structure models) that have significantly broadened the scope of accessible molecular data. Databases such as the AlphaFold Protein Structure Database (Varadi et al., 2024), ESM Metagenomic Atlas (Lin et al., 2023), and ModelArchive (Schwede et al., 2009) encompass nearly a billion predicted structures, representing an increase of three orders of magnitude since structure prediction tools became available. This increase is not just a matter of numbers; it signifies a qualitative enhancement in our capability to access a more comprehensive array of 3D biostructures, many previously unattainable, and form new scientific hypotheses based on them.

The sudden influx of data availability presents dual facets for bioinformaticians: an opportunity for deeper, more comprehensive analysis and a challenge to manage and interpret an overwhelming amount of data (Varadi et al., 2023). One of the primary methods of interacting with these 3D structures is through molecular graphics viewers. Various viewers—such as Mol* Viewer (Sehnal et al., 2021), Jalview (Procter et al., 2021), PyMOL (Rosignoli & Paiardini, 2022), ChimeraX (Meng et al., 2023), and many others, each with unique rendering capabilities—offer diverse perspectives on the same molecular data. The web portals of the Research Collaboratory for Structural Bioinformatics Protein Data Bank (RCSB PDB) and Protein Data Bank in Europe (PDBe) are also supported by molecular viewers that allow users to visually inspect and analyze the details of macromolecular structures (Burley, Bhikadiya, Bi, Bittrich, Chao, et al., 2022; Burley et al., 2023; Varadi et al., 2022). A researcher’s choice of viewer can significantly impact the interpretation and understanding of their structural data.

Each molecular viewer employs distinct techniques and logic for rendering 3D structures, leading to a lack of standardization in visual representation beyond the limited set of commonly used visualization conventions such as cartoons, ribbons, and ball-and-stick representations. This limitation may result in inconsistencies and potential misinterpretation when comparing visualizations across different platforms, underscoring the need for a unified standard in molecular visualization.

In the following protocols, we present MolViewSpec (homepage: https://molstar.org/mol-view-spec/, GitHub project: https://github.com/molstar/mol-view-spec), a novel specification methodology designed to standardize molecular visualizations, thereby dissociating them from the diverse implementation specifics inherent to various molecular viewers. The protocols described here demonstrate the application of MolViewSpec coupled with its Python-based 3D view–building library to craft intricate and tailored 3D visualizations. The protocols cover a range of standard visualization techniques, from cartoon and ball-and-stick models to advanced features like coloring, labeling, and implementing complex transformations such as superposition on macromolecular structure files in formats including PDB, mmCIF, and BinaryCIF. These protocols highlight the strides made towards enhancing the reusability and interoperability of molecular 3D visualizations, a critical development in structural bioinformatics, particularly in large-scale molecular structure analysis. At the moment, MolViewSpec files are only supported by the Mol* 3D viewer. However, the specification was designed to be independent of any particular 3D viewer and nothing prevents other developers from adapting this standard.

In this work, we present three Basic Protocols for creating MolViewSpec views plus two Support Protocols that are useful for processing data needed as input. Basic Protocol 1 showcases the general workflow of the MolViewSpec library and demonstrates its features for loading and visualizing the alignment of two structures. The accompanying Support Protocol 1 provides details on how to compute the required transformation matrix using publicly available tools. Basic Protocol 2 demonstrates a data-driven approach that leverages additional annotation files to customize selections and conveniently add annotations to scenes. The related Support Protocol 2 provides details on how to create annotation files programmatically. Basic Protocol 3 covers global settings, such as changing the background color of the canvas or controlling the overall camera position of the scene.

## BASIC PROTOCOL 1

### CREATING A MOLVIEWSPEC VIEW USING THE MOLVIEWSPEC PYTHON PACKAGE

This protocol describes how the MolViewSpec Python library can be used to show the superposition of two structures: chain A of PDB ID 1oj6 and chain A of PDB ID 5mjd. This example demonstrates basic functionality of the library such as the common protocol of obtaining structure data, parsing said data, specifying which biological assembly to visualize, creating relevant components, and adding representations for components.

All of these steps are modular and can be combined to create complex molecular scenes. The same actions can be applied multiple times to different source data, which effectively allows users to share or reuse snippets between visualization protocols. The modular nature of the library limits the amount of choices at each step and also guides users to the next applicable action as the library is aware of the current context and all possible next steps that are supported at any given time.

The aim of this protocol is to use the MolViewSpec Python library to create a MolViewSpec state file that defines the desired view and then opens this file in the Mol* 3D viewer to reliably and consistently recreate this view.

#### Necessary Resources

Internet connection
Device capable of supporting a web browser, WebGL, and Python
Up-to-date web browser (e.g., Google Chrome, Firefox, Apple Safari) with WebGL support
Python 3.9 or higher with pip or other Python package installer
Text editor (e.g., Gedit, Notepad++) or integrated development environment (IDE, e.g., VSCode)

1. Install the MolViewSpec Python package.
  MolViewSpec is publicly available using PyPI at https://pypi.org/project/molviewspec/. It can be installed locally using package manager (e.g., pip).

      pip install molviewspec

  The expected output is a confirmation that you have successfully installed the most recent version of the MolViewSpec package.
2. Create a Python script.
  Create a script that will hold all relevant code for this protocol. Create a file called “protocol1.py” (see Supporting Information) using a text editor or IDE. Jupyter or Google Colab notebooks are convenient options as well.
3. Import MolViewSpec.
  Import dependencies in the header section of your Python script. For convenience, the molviewspec module is aliased as mvs. All further access should be done in the format

mvs.method().
      import molviewspec as mvs

4. Instantiate a MolViewSpec builder.
  The MolViewSpec builder manages the scene description and can eventually emit JSON format that can reproduce a particular scene. By calling functions, which are provided by the builder, one can gradually add elements to the scene and configure the desired visualization. Thus, it is necessary to instantiate a dedicated MolViewSpec builder for each scene that should be composed.

      builder = mvs.create_builder()

  This code snippet uses the imported MolViewSpec library from step 3, instantiates a new builder instance, and assigns it to a variable called builder, which can be used in subsequent steps. The builder is typed and provides suggestions on applicable actions. Initially, only actions for adding structure data (see step 5) and global properties such as the background color of the canvas are available.
5. Specify the source of structural data.
  The first builder action to take is to specify the source of structural data. Invoke the .download() function exposed by the builder instance and provide the URL of the structural data using the url parameter of the function. In this example, an mmCIF file describing PDB ID 1oj6 is used as the source of structural data. The function returns a new reference of the builder that holds the provided url argument in its internal state and provides a new set of relevant functions that will be explained below (see step 6). This reference is stored in the variable download1.

      download1 = builder.download(url=‘ https://files.wwpdb.org/download/1oj6.cif’)
      download2 = builder.download(url=‘ https://files.wwpdb.org/download/5mjd.cif’)

  It is important to note that the download step and all subsequent steps can be taken multiple times on the builder variable. This allows users to add multiple structures to the same view, and is helpful when visualizing structure alignments or composing complex views with multiple distinct protein structures such as mesoscale models of whole cells. A second structure is added to the builder by invoking the .download() function once more with a different url argument. The resulting builder state is stored in download2, which will be used to perform subsequent actions regarding PDB ID 5mjd.
6. Specify the format of the atomic coordinates file.
  Several established formats exist to exchange structural data. The .parse() function is used to specify which type of atomic coordinates file was provided. Provide the corresponding format via the format parameter. Allowed values are: “mmcif” (mmCIF), “bcif” (BinaryCIF; Sehnal et al., 2020), or “pdb” (legacy PDB format). Both files are in mmCIF format.

      parse1 = download1.parse(format=‘mmcif’)
      parse2 = download2.parse(format=‘mmcif’)

7. Specify which assembly to generate.
  mmCIF files can contain multiple biological assemblies that are identified by a unique identifier. Ordinarily, the first assembly has key “1”, the second assembly has key “2”, and so on. You can generate the desired assembly by using the .assembly_structure() method and passing the corresponding assembly_id as argument.

      structure1 = parse1.assembly_structure(assembly_id=‘1’)
      structure2 = parse2.assembly_structure(assembly_id=‘1’)

  Depending on the use-case, it can also be desired to visualize the asymmetric unit. This can be accomplished by the .model_structure() method, which has no mandatory parameters. The asymmetric unit corresponds to the deposited coordinates in the source file. No instance copies will be generated, which might be the case when requesting biological assemblies.
  More advanced options are also available at this step, such as addressing assemblies or mmCIF data blocks by their index, selecting individual NMR models, or generating crystal lattices by a radius cutoff or Miller indices.
8. *Optional*: Transform coordinates.
  The aim of this protocol is to show the superposition of both structures. Merely loading both files would place both assemblies somewhere in the 3D space. To actually superimpose their atomic coordinates, one structure must be rotated and translated accordingly. An optional transform action can be applied to the result of step 7 using the .transform() function. This function has two optional parameters: rotation (a 9-dimensional vector, representing a 3×3 rotation matrix with column-major or Fortran-style indexing) and translation (a 3D vector). The following arguments for rotation and translation were computed ahead of time using the RCSB PDB Pairwise Structure Alignment application (https://alignment.rcsb.org) (Burley, Bhikadiya, Bi, Bittrich, Chen, et al., 2022; see Support Protocol 1 for details).

      structure2 = structure2.transform(rotation=[-0.39652203922082313, 0.918022802798312,
         0.002099036562725462, 0.9068461182538327, 0.39133670281585825, 0.1564790811487865,
         0.14282993460796656, 0.06395090751149791, -0.9876790426086504],
         translation=[-17.636085896690037, 7.970761314734439, 88.54613248028247])

  Note that this step is only applied to the second structure (PDB ID 5mjd) to transform its position in 3D with respect to the first structure (PDB ID 1oj6).
9. Create components of interest.
  All steps to this point have prepared the visualization of 3D structure data. Next, the components need to be defined. Components are the result of selections of varying granularity. They can describe a whole protein structure, an individual chain, an individual amino acid residue via its sequence position, or even individual atoms.
  Components are created by invoking the .component() method provided by the returned object of steps 7–8. This method has a single optional parameter, selector. If the selector parameter is not specified, the whole structure will be selected and represented as a single component. The same behavior can be achieved by calling .component(selector=‘all’). The following snippet selects polymer chains from both structures and groups them into a component. Furthermore, ligands are selected and grouped into a distinct component.

      polymer1 = structure1.component(selector=‘polymer’)
      ligand1 = structure1.component(selector=‘ligand’)
      
      polymer2 = structure2.component(selector=‘polymer’)
      ligand2 = structure2.component(selector=‘ligand’)

  Built-in selectors include: “all”, “polymer”, “protein”, “nucleic”, “branched” (for oligosaccharides), “ligand”, “ion”, and “water”.
10. Create representations for components.
  Components can then be used to create representations, which are things that will be rendered on the canvas. The following representations are supported: “cartoon”, “surface” (molecular surface), and “ball_and_stick”. Similar to the previous step, the method can be invoked without passing any arguments. In that case, the builder will default to a cartoon representation. In our example, polymeric components from both structures will be represented by a cartoon stylization, whereas ligands will be depicted as ball-and-stick.

      polymer_representation1 = polymer1.representation() # default: ‘cartoon’
      ligand_representation1 = ligand1.representation(type=‘ball_and_stick’)

      polymer_representation2 = polymer2.representation(type=‘cartoon’)
      ligand_representation2 = ligand2.representation(type=‘ball_and_stick’)

11. *Optional*: Adjust color of representations.
  Lastly, it is helpful in structure alignments to assign distinct colors to all structures to help distinguish them visually. The representation variables provide a .color() method, which has a single color parameter that sets the color. Colors should be provided in their hex representation. Any of the recognized SVG color names (https://www.w3.org/TR/SVG11/types.html#ColorKeywords) are supported as well.

      polymer_representation1.color(color=‘#e19039’) # orange
      ligand_representation1.color(color=‘#eec190’) # desaturated orange

      polymer_representation2.color(color=‘#4b7fcc’) # blue
      ligand_representation2.color(color=‘#9cb8e3’) # desaturated blue

  There is no need to store the result of these function calls in variables as this resembles the deepest level of nesting. No more fine-grained actions are supported.
  In general, the .color() method returns a reference to the representation and can streamline the specification of multiple colors for different selections by chaining operations.
12. Export the builder state.
  At this point, the builder instance is fully populated with all data sources, parsing information, and details on how to depict a structural alignment of both structures. In order to pass this information to a local file or a compatible 3D viewer, the .get_state() method of the builder variable can be used. The following statement acquires the state of the MolViewSpec builder in MVSJ format (which is a JSON representation of the state with additional metadata) and prints it to the console using the print() function. By default, the resulting JSON will be indented by two whitespaces, but this can be configured by passing an integer using the indent parameter.

      print(builder.get_state())

**Figure 1:**
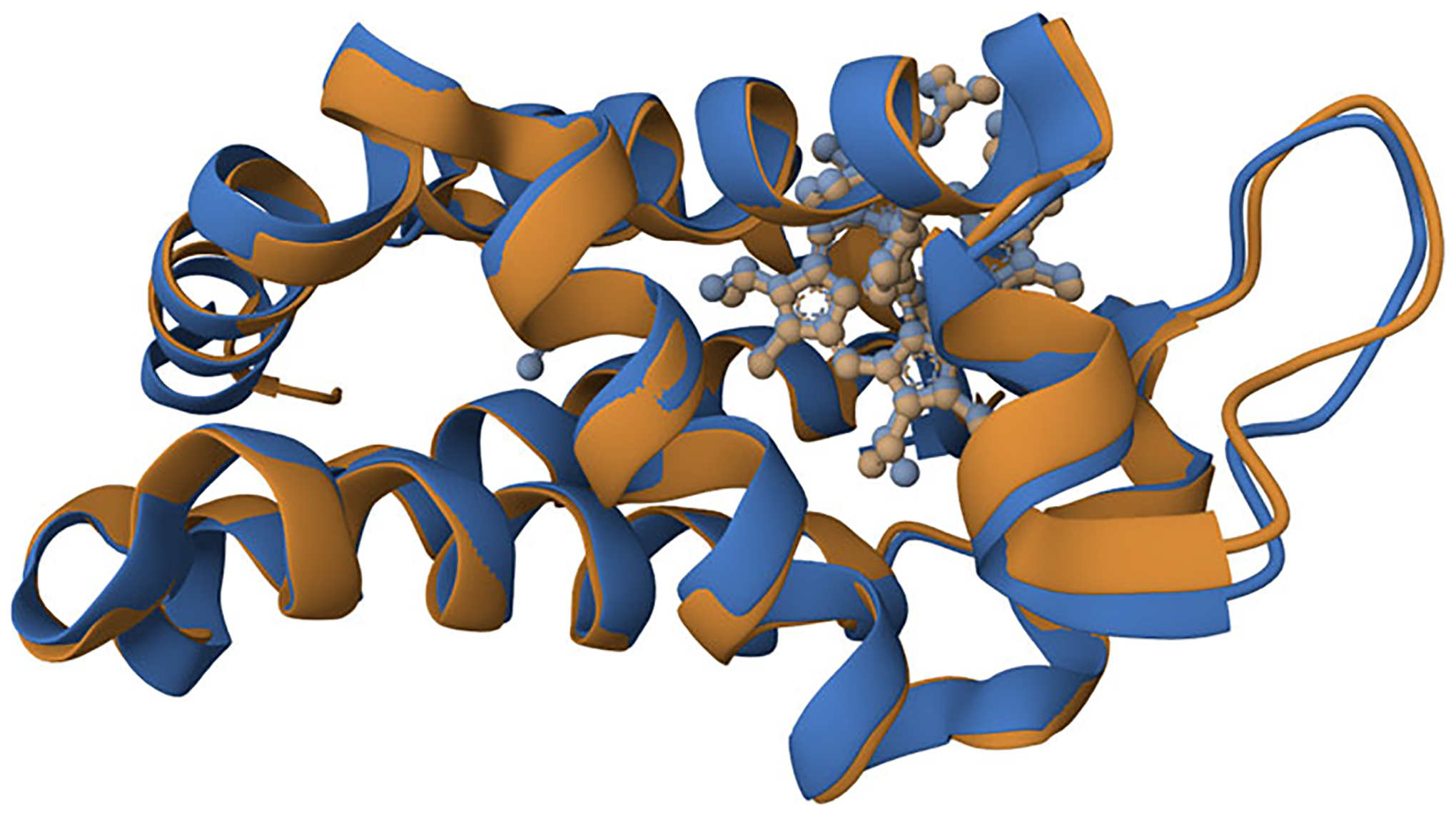
Result of Basic Protocol 1. Chain A of PDB ID 1oj6 (orange) is aligned with chain A of PDB ID 5mjd (blue) in cartoon representation. Heme ligands are shown in ball-and-stick representation.

  This console output can be stored in a file called “protocol1.mvsj” (see Supporting Information) and captures the complete builder state that was modularly composed by all previous steps. Save this file in your local file system to persist your results. We refer to this file as MolViewSpec state file.
13. Open MolViewSpec file in a compatible 3D viewer.
  Drag and drop the “protocol1.mvsj” file from step 12 into an instance of the Mol* 3D viewer (e.g., https://molstar.org/viewer/). Mol* will recognize the file format and create the scene described by the MVSJ file, as seen in Figure 1 and at this link. The resulting view shows chain A of PDB ID 1oj6 in orange superimposed with chain A from PDB ID 5mjd in blue.

## BASIC PROTOCOL 2

### CREATING A MOLVIEWSPEC VIEW WITH REFERENCE TO MOLVIEWSPEC ANNOTATION FILES

This protocol describes how the MolViewSpec Python library can be used to build molecular views that reference additional data resources known as MolViewSpec annotation files. This data-driven visualization approach allows us to separate the view description from the actual data (e.g., colors assigned to individual residues), resulting in better code and data reusability. This protocol describes how to build the view description (i.e., the MolViewSpec state file), assuming we already have our data prepared in MolViewSpec annotation files. For the process of creating the MolViewSpec annotation files themselves, see Support Protocol 2.

All the steps in this protocol can be freely combined with any steps described in Basic Protocol 1 to build custom views. For example, one can define components in the regular way using the .component() method and then apply colors based on MolViewSpec annotation using .color_from_uri().

#### Necessary Resources

Internet connection
Device capable of supporting a web browser, WebGL, and Python
Up-to-date web browser (e.g., Google Chrome, Firefox, Apple Safari) with WebGL support
Python 3.9 or higher with pip or other Python package installerFiles
MolViewSpec annotation files. In this example, we use “annotations-1h9t.cif”; see Supporting Information; see Support Protocol 2 for more details.

1. Install the MolViewSpec Python package, create a script, instantiate builder, and specify structural data.
  The first few steps of this protocol are in essence the same as in Basic Protocol 1. Perform the setup and build the view description down to the structure level as described there (see Basic Protocol 1, steps 1–7). After performing these steps, our Python script “protocol2.py” (see Supporting Information) might look like this:

      import molviewspec as mvs

      builder = mvs.create_builder()

      structure = (builder
                     .download(url=‘https://files.wwpdb.org/download/1h9t.cif’)
                     .parse(format=‘mmcif’)
                     .model_structure()
                     )

2. Create components using MolViewSpec annotations.

      protein = structure.component_from_uri(
            uri=‘./annotations-1h9t.cif’, format=‘cif’,
            block_header=‘1h9t_annotations’, category_name=‘components’,
            field_name=‘component’, field_values=‘Protein’, schema=‘chain’)
      dna = structure.component_from_uri(
            uri=‘./annotations-1h9t.cif’, format=‘cif’,
            category_name=‘components’, field_values=‘DNA’, schema=‘chain’)
      ions = structure.component_from_uri(
            uri=‘./annotations-1h9t.cif’, format=‘cif’,
            category_name=‘components’, field_values=[‘Gold’, ‘Chloride’],
            schema=‘chain’)

  Data-driven structure components can be created using the .component_from_uri() method. Provide the location (uri) and format of the MolViewSpec annotation file in which the components are defined. This snippet uses a relative URI “./annotations.cif”, which will be resolved against the URI of the MVSJ file itself, e.g., if we serve the MVSJ file at “https://example.org/view.mvsj”, then the annotation file will have to be at “https://example.org/annotations-1h9t.cif”. The value of the format parameter can be “cif”, “bcif”, or “json”, depending on the format of the annotation file.
  Parameters block_header and category_name are used to indicate a specific block and category in the annotation file (only applicable for annotation files in CIF or BinaryCIF format). These parameters can be omitted, indicating the first category of the first block.
  Parameters field_name and field_values are used to specify a subset of annotation rows that should be included in the created component. Namely, field_name indicates one of the columns in the annotation table, and field_values indicates the values in that column to be included (field_name can be omitted if the column name is “component”). Omitting these parameters indicates that the whole table should be included in the component.
  Finally, the schema parameter defines the granularity of the created component. In our example, schema=‘chain’ means that we are selecting whole chains and the component is defined by columns “label_asym_id” and “label_entity_id” in the table (detailed information on mmCIF fields can be found at https://mmcif.wwpdb.org/dictionaries/mmcif_pdbx_v50.dic/Index/). Use the schemas “entity”, “chain”, “residue”, “residue_range”, and “atom” to select components based on the mmCIF numbering (columns prefixed with “label_”); use “auth_chain”, “auth_residue”, “auth_residue_range”, and “auth_atom” to select components based on author-provided numbering (columns prefixed with “auth_”). The wildcard schema “all_atomic” can be used to take into account all columns present in the annotation table.
3. Create representations.

      protein_repr = protein.representation(type=‘cartoon’)
      dna_repr = dna.representation(type=‘ball_and_stick’)
      ions_repr = ions.representation(type=‘surface’)

**Figure 2:**
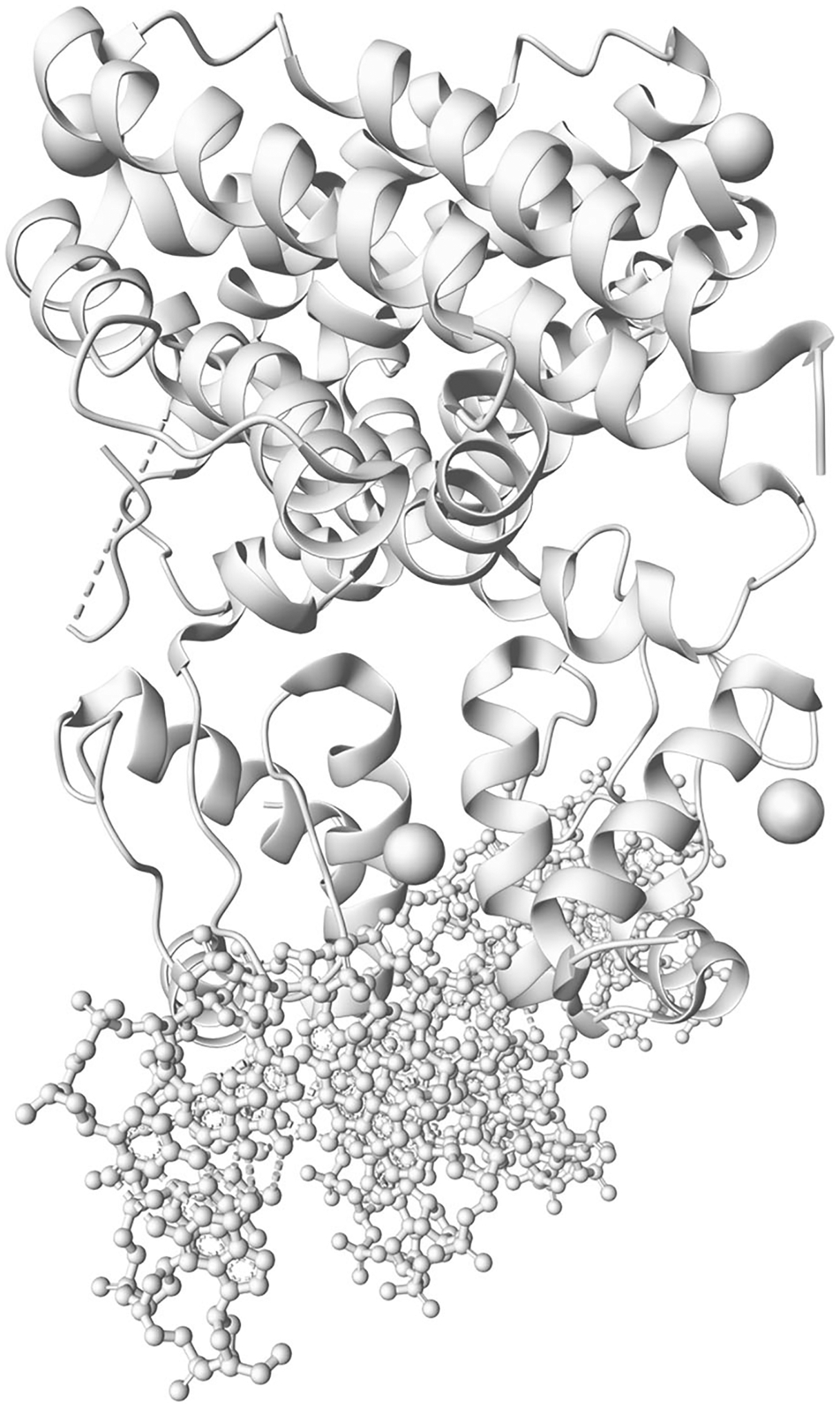
Result of Basic Protocol 2 after step 3. Loaded representations for components in 1h9t (visualized by Mol* Viewer).

  Creating representations is achieved by the .representation() method, regardless of whether the component was created by .component() or .component_from_uri(). The result of this step is shown in Figure 2.
4. Apply coloring using MolViewSpec annotations.

      protein_repr.color_from_uri(
            uri=‘./annotations-1h9t.cif’, format=‘cif’,
            block_header=‘1h9t_annotations’, category_name=‘annotations’,
            field_name=‘color’, schema=‘residue_range’)
      dna_repr.color_from_uri(
            uri=‘./annotations-1h9t.cif’, format=‘cif’,
            category_name=‘annotations’, schema=‘residue_range’)
      ions_repr.color_from_uri(
            uri=‘./annotations-1h9t.cif’, format=‘cif’,
            category_name=‘annotations’, schema=‘residue_range’)

**Figure 3:**
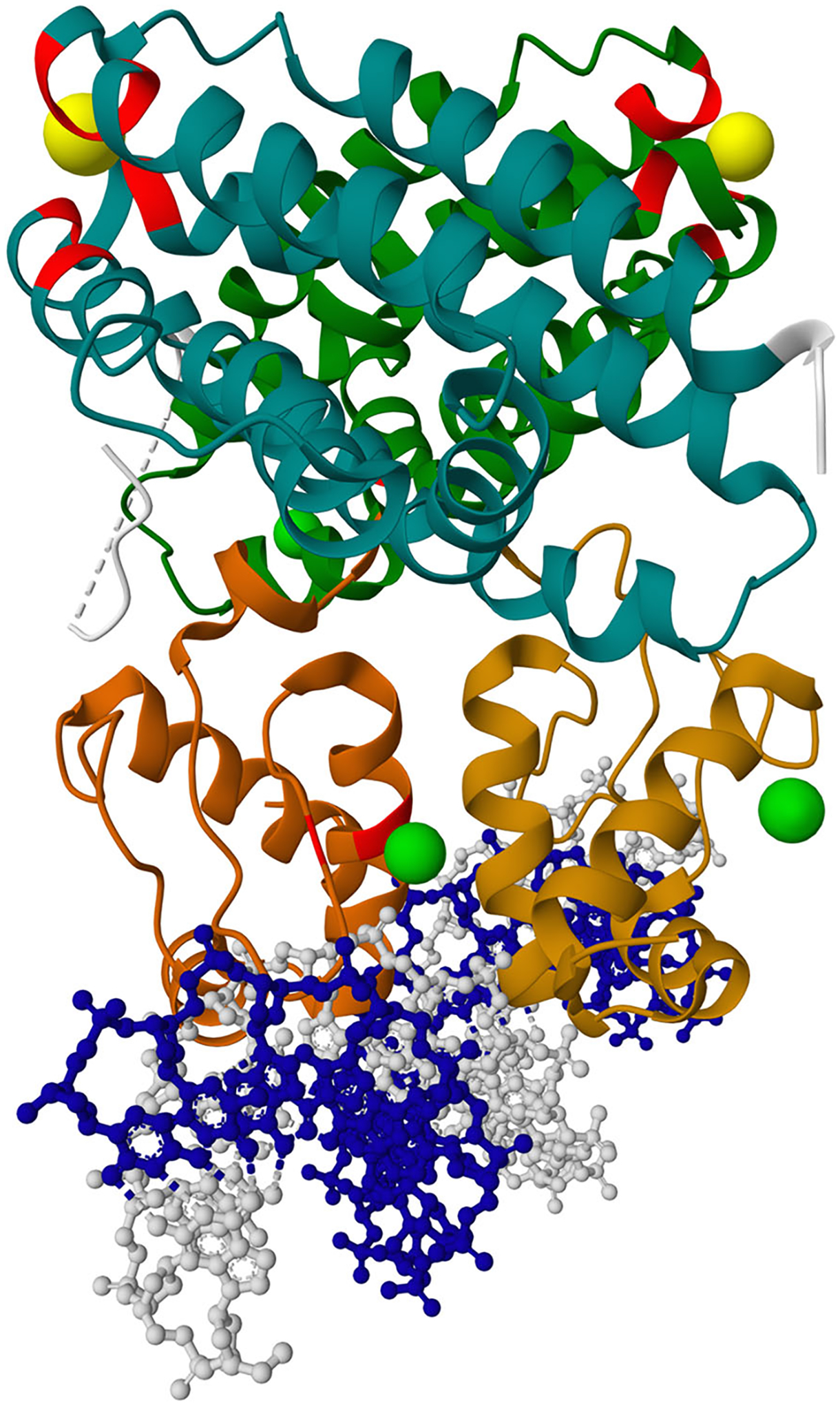
Result of Basic Protocol 2 after step 4. Applied colors from MolViewSpec annotations (visualized by Mol* Viewer).

  Data-driven coloring can be added to a representation via the .color_from_uri() method.
  The meaning of parameters uri, format, block_header, category_name, and schema is the same as for .component_from_uri().
  Parameter field_name indicates the column that holds the color values (can be omitted if the column name is “color”). The color values in the annotation file should be in the same format as the colors used in the MolViewSpec state file (i.e., either a name like orange or a hex code like #e19039).
  If multiple annotation rows apply to the same part of the structure, this part will in the end be colored by the last applied color. For instance, if the first row applies red to residues 1–100 and the second row applies blue to residues 100–120, then residue 100 will be blue. The same logic applies when multiple colors are applied to the same representation (e.g., protein_repr.color(color = ‘gray’). color_from_uri(…)). The result of this step is shown in Figure 3.
5. Add labels using MolViewSpec annotations.

      structure.label_from_uri(
            uri=‘./annotations-1h9t.cif’, format=‘cif’,
            block_header=‘1h9t_annotations’, category_name=‘annotations’,
            field_name=‘label’, schema=‘residue_range’)

**Figure 4:**
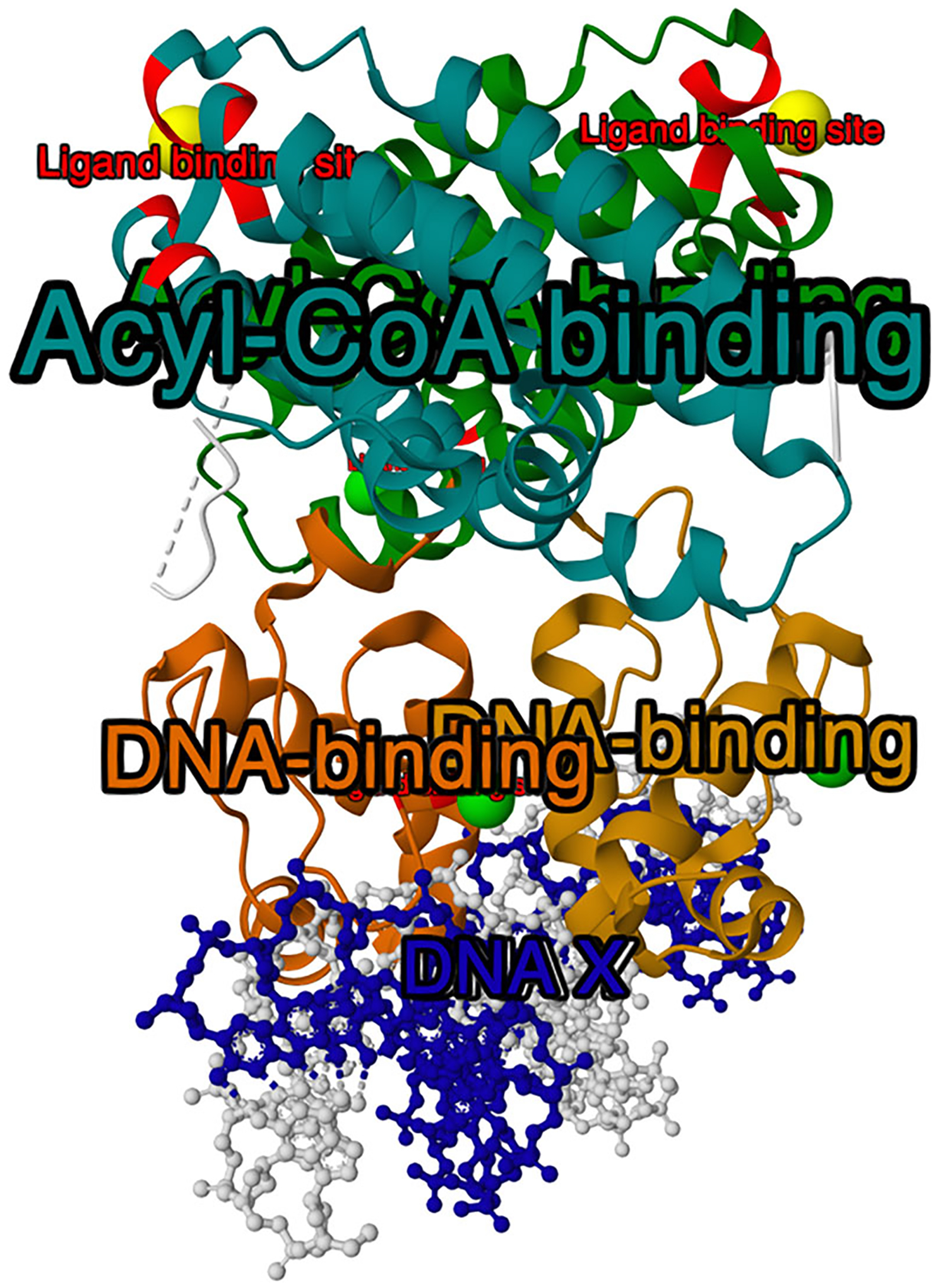
Result of Basic Protocol 2 after step 5. Added labels from MolViewSpec annotations (visualized by Mol* Viewer).

  Data-driven labels can be added to a structure via the .label_from_uri() method.
  The meaning of parameters uri, format, block_header, category_name, and schema is the same as for .component_from_uri().
  Parameter field_name indicates the column that holds the label values (can be omitted if the column name is “label”).
  By default, each annotation row results in one label in the 3D view. However, if the annotation table contains a column named “group_id”, this column will be used to group rows with the same group_id value and apply only one label per group. Rows with missing group_id (represented in CIF by the special value “.”) behave as separate groups. The result of this step is shown in Figure 4.
6. Add tooltips using MolViewSpec annotations.

      structure.tooltip_from_uri(
            uri=‘./annotations-1h9t.cif’, format=‘cif’,
            block_header=‘1h9t_annotations’, category_name=‘annotations’,
            field_name=‘label’, schema=‘residue_range’)

**Figure 5:**
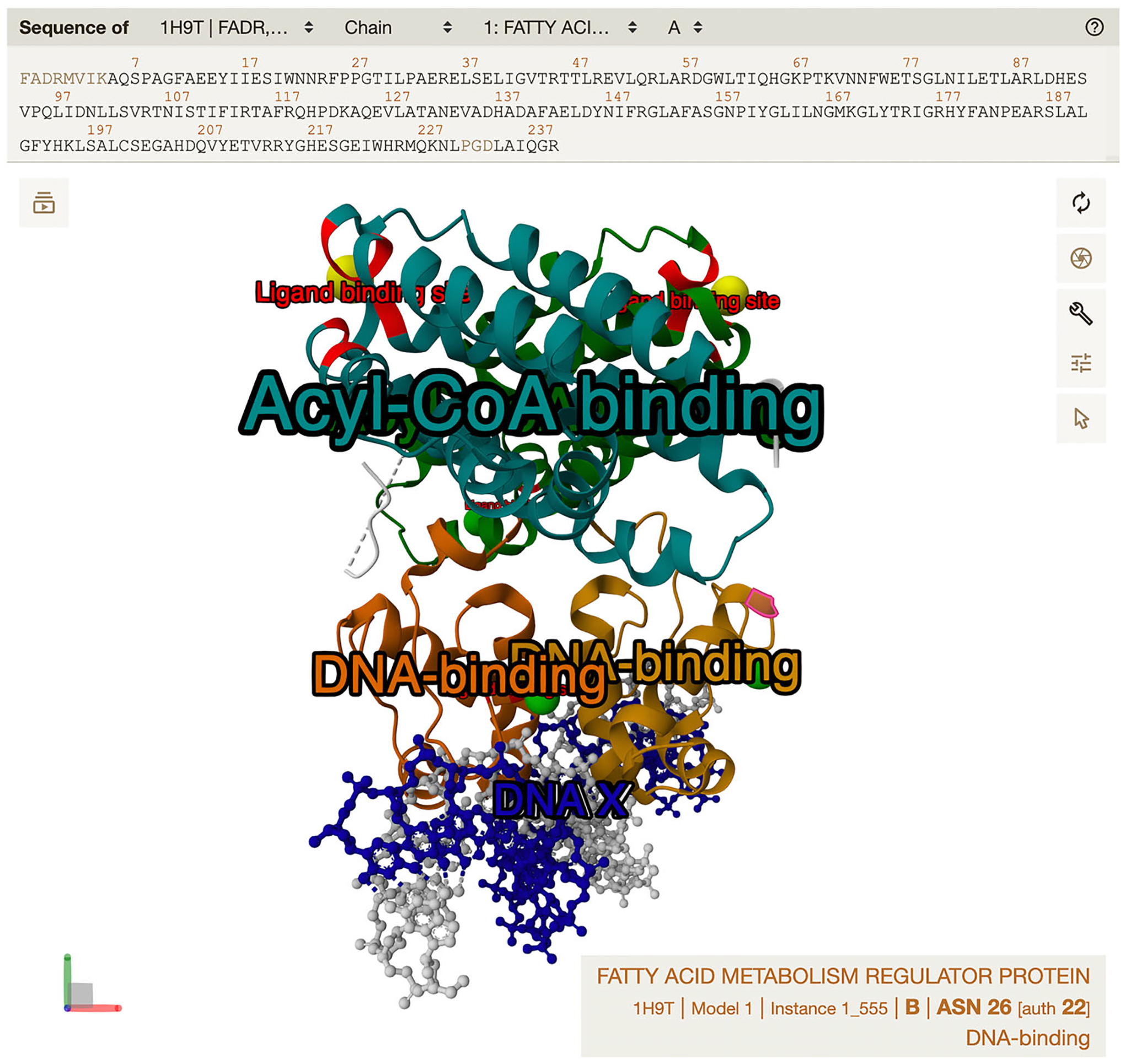
Result of Basic Protocol 2 after step 6. Added tooltips from MolViewSpec annotations are shown in the interface of Mol* Viewer. The tooltip text “DNA-binding” is visible in the bottom right corner when hovering over the respective part of the structure.

  A tooltip, as opposed to a label, refers to text that is not an integral part of the visualization but is presented to users when they interact with a structure component as shown in Figure 5. The exact behavior can vary between viewers, but typically the tooltip will be shown somewhere on the screen when the user hovers over the component. In some contexts (e.g., when rendering static images), tooltips do not apply at all.
  Data-driven tooltips can be added to a structure via the .tooltip_from_uri() method.
  The meaning of parameters uri, format, block_header, category_name, and schema is the same as for .component_from_uri().
  Parameter field_name indicates the column that holds the tooltip values (can be omitted if the column name is “tooltip”). In our example, we take the tooltips from the same column as the labels.
7. Export the builder state.

      builder.save_state(destination=‘protocol2.mvsj’,
                        title=‘An example with MVS annotations’, indent=2)

  The created state can be exported in JSON-based MVSJ format using the methods .get_state() (to get as a string) and .save_state() (to save into a file). The format can be customized by the optional indent parameter, and additional information can be saved with the state by the optional title, description, and description_format parameters.
8. Ensure availability of the annotation files.
  At this stage, we must ensure that the referenced annotation files are available to the viewer. There are multiple scenarios, as the URI reference can be either absolute or relative and can use various protocols.
  - The annotation file URI uses “http(s)” scheme (or the annotation file URI reference is relative and the state file URI uses “http(s)” scheme, i.e., the state file is served from the web). To achieve this, you must host your annotation file on a server and make it publically available. Also make sure CORS is enabled for the hosted files.
  - The annotation file URI uses “file” scheme (or the annotation file URI reference is relative and the state file URI uses “file” scheme, i.e., the state file is loaded from disk). This is applicable for desktop viewers and command-line applications that have access to the disk (such as the Mol* command-line utility mvs-render). It is not applicable for web viewers because, even when the state file is loaded from the disk (via either a menu or drag-and-drop), the browser does not allow access to other files on the disk for security reasons. Therefore, dragging and dropping the state file into an instance of the Mol* 3D viewer (see Basic Protocol 1, step 13) will not work in this case.
  - The annotation file URI reference is relative and the state file with the annotation file are packed together in an MVSX archive. Technically, an MVSX archive is simply a ZIP archive containing the state file named as “index.mvsj” and any number of other files (can be MVS annotation files but also structure files). The extension .mvsx is used to distinguish it from a regular .zip archive, but the structure is the same. Therefore, it is very easy to create via a Python script:

      import zipfile

      with zipfile.ZipFile(‘protocol2.mvsx’, mode=‘w’) as z:
             z.write(‘protocol2.mvsj’, arcname=‘index.mvsj’)
             z.write(‘annotations-1h9t.cif’, arcname=‘annotations-1h9t.cif’)

    In this case, the relative URI reference in the state file will resolve to the other file stored in the archive. Thanks to this, the MVSX archive can simply be loaded into Mol* Viewer by dragging and dropping, and can also be served from the web.
9. Open MolViewSpec file in compatible 3D viewer.
  Open the created state file, e.g., drag and drop the MVSX file into an instance of the Mol* 3D viewer or go to https://molstar.org/viewer/?mvs-format={FORMAT}&mvs-url={URL}, where {URL} specifies the URL where the state file is hosted and {FORMAT} specifies the format of the state file (mvsj/mvsx). The resulting view is shown in Figure 5 and at this link: https://molstar.org/viewer/?mvs-format=mvsj&mvs-url=https%3A%2F%2Fmolstar.org%2Fmol-view-spec%2Fexamples%2Fannotations%2Fstate.mvsj&hide-controls=1.
  *NOTE*: Thanks to the structure of the CIF (and BinaryCIF) format, a single file can accommodate both structural data and MolViewSpec annotations. To reference these annotations, you can use component_from_source, color_from_source, label_from_source, and tooltip_from_source methods, which behave in the same way as their _from_uri counterparts but load the annotation from the same CIF (or BinaryCIF) file from which the structural data are loaded. These methods also take the same parameters, except for uri and format, which are obviously not necessary.

## BASIC PROTOCOL 3

### CREATING A MOLVIEWSPEC VIEW WITH LABELS AND OTHER ADVANCED FEATURES

This protocol describes more advanced features of the MolViewSpec Python library such as assigning labels to components, adding tooltips, and specifying global canvas properties (e.g., background color and custom camera orientation). The aim of this protocol is to use the MolViewSpec Python library to create a more detailed JSON file that defines the desired view and then open this file in the Mol* 3D viewer to reliably and consistently recreate this view.

#### Necessary Resources

Internet connection
Device capable of supporting a web browser, WebGL, and Python
Up-to-date web browser (e.g., Google Chrome, Firefox, Apple Safari) with WebGL support
Python 3.9 or higher with pip or other Python package installer

1. Perform common MolViewSpec actions to create empty structure.
  Create a standard MolViewSpec scene as described (see Basic Protocol 1, steps 1–7). The condensed “protocol3.py” Python code (see Supporting Information) will look like this:

      import molviewspec as mvs

      builder = mvs.create_builder()
      # note: updated CIF from PDBe provides explicit bond information to render nicely
      download = builder.download(url=‘ https://www.ebi.ac.uk/pdbe/entry-files/download/4hhb_updated.cif’)
      parse = download.parse(format=‘mmcif’)
      structure = parse.assembly_structure(assembly_id=‘1’)

  This will load PDB ID 4hhb and create a structure object for its first biological assembly.
2. Create a custom component expression.
  When selections will be reused, it can make sense to store them in a variable. Here, the iron atom (with “label_atom_id” = “FE”) from the first heme ligand (“label_asym_id” = “E”) is selected and assigned to the fe_selector variable.

      fe_selector = mvs.ComponentExpression(label_asym_id=‘E’, label_atom_id=‘FE’)

  The ComponentExpression class supports a plethora of properties that enable the selection of individual atoms, residues, chains, or entities.
3. Create a representation for the first heme ligand.
  In this protocol, the first heme ligand of PDB ID 4hhb should be visualized. The ligand can be uniquely identified by its “label_asym_id” as defined in the mmCIF source file.

      ligand = structure.component(selector=mvs.ComponentExpression(label_asym_id=‘E’))
      ligand.representation(type=‘ball_and_stick’).color(color=‘#ffffff’).color(selector=fe_
         selector, color=‘#ff4500’)

  The ligand variable now holds a reference to a component describing the first heme ligand. In the second line, a ball-and-stick representation in white color is added. Lastly, the .color() method is used to change the color of the previously selected iron ion (referenced by the fe_selector variable) to an orange-red color.
4. Add labels and tooltips to components.
  Custom text labels can be defined and will appear close to the selected element in 3D. The fe_selector variable is used to create another component that captures only the FE atom of the first heme ligand. The .label() method adds a text label to components. Its content can be defined using the text parameter. Analogously, the .tooltip() method assigns custom tooltips to components. These tooltips appear in the bottom-right corner of the Mol* 3D viewer if hovering over the corresponding element, providing a way of showing more detailed information.
  *NOTE*: The selection mode must be changed to view the tooltip when hovering over the iron ion. In Mol*, this can be achieved by clicking the mouse cursor icon (labeled “Toggle Selection Mode”) located in the top-right corner of the canvas. Subsequently, adjust the value of the newly revealed “Picking Level” dropdown menu from “Residue” to “Atom/Coarse Element”.

      fe = structure.component(selector=fe_selector)
      fe.label(text=‘Iron Ion’).tooltip(text=‘Additional info that appears only on mouseover’)

5. Specify global properties: Background color.
  The builder supports a global .canvas() action that enables setting the background color, e.g., to a light orange.

      builder.canvas(background_color=‘#ffcf6b’)

  Colors can be provided in hex representation or as SVG color names (https://www.w3.org/TR/SVG11/types.html#ColorKeywords).
6. Specify global properties: Camera position.
  The builder supports another global action via the .camera() method. This method has two parameters: target and position, which are both 3D vectors. target supplies the coordinates of the object to be viewed, whereas position defines the location of the camera. A third optional parameter is up, another 3D vector that controls what appears in the upper part of the canvas.

      # camera to look at heme ligand, orthogonal to porphyrin group
      builder.camera(target=(18, 18, 24), position=(30 , 12, 25))

**Figure 6:**
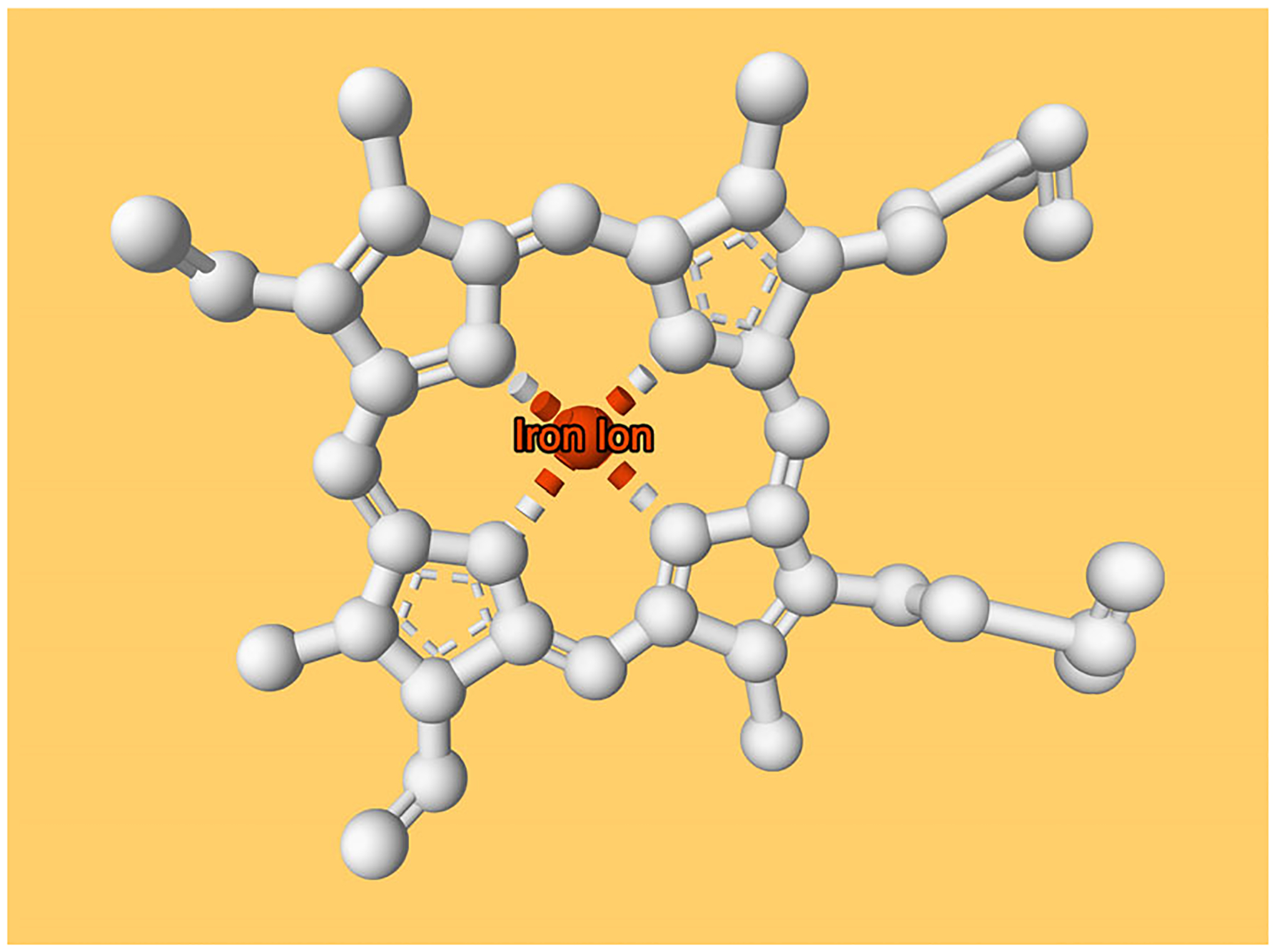
Result of Basic Protocol 3. Ball-and-stick representation of first heme ligand (chain E) of PDB ID 4hhb. This protocol employs more advanced features, such as changing background color, positioning the camera, and assigning labels.

  Viewers implementing MolViewSpec will use their own protocol to orient the camera relative to the visualized content. Truly reproducible views can only be defined by explicitly setting the camera position.
  Another way of controlling the camera position is to use the .focus() method, which is exposed by all components. Invoking .focus() will position the camera so that all elements of that selection are visible.
7. Export this view and open it in a compatible 3D viewer.
  Export the state of the builder variable as JSON format with file name “protocol3.mvsj” (see Supporting Information) as described (see Basic Protocol 1, steps 12–13) and open it, e.g., by dragging and dropping it into an instance of the Mol* 3D viewer. The resulting view is shown in Figure 6 and at this link. It shows a single heme ligand of PDB ID 4hhb. The ligand is displayed in white, the coordinated iron ion is in orange-red and labeled with custom text, and the background color is set to orange. The camera is positioned so that it looks directly at the heme ligand from above, orthogonal to the porphyrin group.

## SUPPORT PROTOCOL 1

### COMPUTING ROTATION AND TRANSLATION VECTORS

MolViewSpec supports structure alignments but requires users to provide appropriate rotation and translation vectors that describe how the atomic coordinates of a structure should be transformed. This protocol suggests one way to compute these values using publicly available resources and showcases how to use the result in a MolViewSpec file. Our example aligns chain A of 1oj6 with chain A of 5mjd, but the approach works for any identifier registered in the PDB archive or AlphaFold DB plus arbitrary structure data if uploaded to a URL.

#### Necessary Resources

PC with text editor (e.g., Gedit, Notepad++) or integrated development environment (IDE, e.g., VSCode)

1. Obtain alignment using the RCSB PDB Pairwise Structure Alignment application.
  Navigate to the application (https://www.rcsb.org/alignment) and specify the two inputs: “1OJ6” and “5MJD”. This will populate the inputs for “Chain ID” and “Begin” and “End” positions appropriately. Make sure to select the correct chain if chains other than the first one should be aligned. Click the “Compare” button to compute and visually validate the alignment.
2. Obtain a 4×4 transformation matrix from the alignment response.
  Click the “Alignment API” button in the top-right corner of the user interface to access the underlying application programming interface (API). This page will show the API request made to align both entries. You will also see the API response after clicking the play icon in the header bar of the user interface.
  The API response contains a property called transformations, an array that contains the 4×4 transformation matrix that will move the atomic coordinates of an entry appropriately. The first matrix in the array is always the identity matrix because coordinates of the reference structure will be fixed. The second matrix contains the transformation that will superimpose the second structure onto the first. In this example, the 4×4 matrix looks like this:

      [-0.3965220392, 0.9180228028, 0.0020990366, 0, // values 1…4
      0.9068461183, 0.3913367028, 0.1564790811, 0, // values 5…8
      0.1428299346, 0.0639509075, -0.9876790426, 0, // values 9…12
      -17.6360858967, 7.9707613147, 88.5461324803, 1] // values 13…16

3. Convert to rotation and translation vectors.
  The rotation vector is captured by values 1, 2, 3, 5, 6, 7, 9, 10, and 11, whereas values 13, 14, and 15 describe the translation vector. Values in the fourth column can be ignored.
  The obtained values can then be used in the following fashion:

      structure2 = structure2.transform(rotation=[-0.39652203922082313, 0.918022802798312,
         0.002099036562725462, 0.9068461182538327, 0.39133670281585825, 0.1564790811487865,
         0.14282993460796656, 0.06395090751149791, -0.9876790426086504],
         translation=[-17.636085896690037, 7.970761314734439, 88.54613248028247])

## SUPPORT PROTOCOL 2

### CREATING A MOLVIEWSPEC ANNOTATION FILE

MolViewSpec annotations are used to define data for substructures (components), colors, labels, and tooltips separately from the view description (state file). The state file can then reference the annotation files instead of including all of the data. A state file can combine references to many annotation files, and an annotation file can be referenced in many state files, thus providing modularity and data reusability. This protocol describes how to create a MolViewSpec annotation file; Basic Protocol 2 describes how to reference it in a state file.

MolViewSpec currently allows three formats for encoding the annotations: CIF, BCIF (BinaryCIF), and JSON. This protocol uses the CIF format, a table-based format that is commonly used in structural biology to store structures or any kind of tabular data (Evans et al., 2021). A CIF file is divided into one or more blocks, each containing one or more categories. In the context of annotations, a category represents an annotation table and consists of columns (fields) and rows. BCIF format has the same internal structure as CIF but uses different encoding that results in much smaller file sizes, and is therefore the optimal choice when working with large data (Sehnal et al., 2020). The third alternative is the JSON format. Its advantage is that most programming languages provide support for reading and writing JSON. However, it lacks the block-category structure, so it can only store a single annotation table. In Supporting Information, we provide the same set of annotations encoded with all three formats (see “Annotations-1h9t-components.json”, “Annotations-1h9t.bcif”, “Annotations-1h9t.cif”, and “Annotations-1h9t-annotations.json”).

#### Necessary Resources

PC with text editor (e.g., Gedit, Notepad++) or integrated development environment (IDE, e.g., VSCode)

1. Create a CIF file.
  Create an empty text file named “annotation-1h9t.cif”, which will hold the annotations. Open the file in a text editor or IDE.
2. Open a block.
  Blocks in a CIF file are marked by a data_ directive immediately followed by the name of the block (block header). In our case, the block header will be “1h9t_annotations”:

      data_1h9t_annotations

3. Open a category and define the columns.
  Within the block, each category (annotation table) is typically marked by a loop_ directive followed by the list of column names in the format _{CATEGORY_NAME}.{COLUMN_NAME}:

      loop_
      _components.label_asym_id
      _components.component

  This snippet opens a category named “components” with two columns named “label_asym_id” and “component”.
  MolViewSpec annotations distinguish two types of columns. Independent variable columns are used to select substructures; dependent variable columns assign values such as color or label to these substructures. There is a predefined list of independent variable columns (label_entity_id, label_asym_id, label_seq_id, beg_label_seq_id, end_label_seq_id, label_atom_id, auth_asym_id, auth_seq_id, pdbx_PDB_ins_code, beg_auth_seq_id, end_auth_seq_id, auth_atom_id, type_symbol, atom_id, atom_index, group_id). Dependent variable columns can have any name.
  In our example, “label_asym_id” is an independent variable column (selects substructures based on the chain identifier) and “component” is a dependent variable column (assigns component name).
4. Add annotation rows into the category.
  Any number of annotation rows can be added. Each row must list values for each column separated by spaces.

      A Protein
      B Protein
      C DNA
      D DNA
      E Gold
      H Gold
      F Chloride
      G Chloride
      I Chloride

  This example defines four components: “Protein” consisting of chains A, B; “DNA” with chains C, D; “Gold” with chains E, H; and “Chloride” with chains F, G, I.
5. *Optional*: Add more categories.
  A new category can be opened by using a loop_ directive again and defining the column names and annotation rows for the new table. Category names must be unique within the block.

      loop_
      _annotations.label_asym_id
      _annotations.beg_label_seq_id
      _annotations.end_label_seq_id
      _annotations.color
      _annotations.label
      _annotations.group_id
      A       9     83   ‘#dd6600’   ‘DNA-binding’             .
      A      84   231   ‘#008800’   ‘Acyl-CoA binding’        .
      B       9     83   ‘#cc8800’   ‘DNA-binding’           .
      B      84   231   ‘#008888’   ‘Acyl-CoA binding’       .
      C       .       .   ‘#1100aa’   ‘DNA X’                 .
      D       .       .   ‘#dddddd’   ‘DNA Y’                 .
      E       .       .   ‘#ffff00’   ‘Gold’                   .
      H       .       .   ‘#ffff00’   ‘Gold’                   .
      F       .       .   ‘#00dd00’   ‘Chloride’              .
      G       .       .   ‘#00dd00’   ‘Chloride’              .
      I       .       .   ‘#00dd00’   ‘Chloride’              .
      A      57    57   ‘red’          ‘Ligand binding site’   1
      A      67    67   ‘red’          ‘Ligand binding site’   1
      A    121   121   ‘red’          ‘Ligand binding site’   2
      A    125   125   ‘red’          ‘Ligand binding site’   2
      A    129   129   ‘red’          ‘Ligand binding site’   2
      A    178   178   ‘red’          ‘Ligand binding site’   3
      A    203   205   ‘red’          ‘Ligand binding site’   2
      B    121   121   ‘red’          ‘Ligand binding site’   4
      B    125   125   ‘red’          ‘Ligand binding site’   4
      B    129   129   ‘red’          ‘Ligand binding site’   4
      B    203   205   ‘red’          ‘Ligand binding site’   4

  In this category, the independent variable columns (label_asym_id, beg_label_seq_id, end_label_seq_id) select substructures based on the chain identifier and residue range, while the dependent variable columns assign color and label. The special column “group_id” groups annotation rows together to create more complex selections for application of labels. For example, this annotation table creates three separate labels saying “Chloride” (one per row), but only four labels saying “Ligand binding site” (eleven rows are grouped into four groups).
  This example also demonstrates how quotes can be used to include spaces and special characters within string values (e.g., ‘Ligand binding site’, ‘#dd6600’). A value can also be omitted using a period (.), e.g., the “DNA X” label is applied to the whole chain C because the residue numbers are omitted. Here use multiple spaces between values to align the columns, but this is just for visual clarity; using a single space is sufficient. More details about the CIF syntax can be found in its documentation (https://mmcif.wwpdb.org/).
6. *Optional*: Add more blocks.
  At this point we can add more blocks, each started with a data_ directive and the block header. Block headers must be unique within the whole file.

## COMMENTARY

### Background Information

MolViewSpec offers a standardized mechanism for describing 3D molecular visualizations, significantly increasing the reproducibility of sophisticated interactive renderings. A declarative, data-driven approach powers the comprehensive definition and visualization of biomolecular entities. MolViewSpec supports standardized 3D representation types, diverse coloring schemes, and associated annotations covering structural, biological, and functional data (Table 1). The 3D molecular graphics viewer Mol* interprets the MolViewSpec format, enhancing the accessibility and interpretability of complex molecular data for researchers, educators, and students.

### Critical Parameters

The key parameters for instantiating a new visualization are .download(url=) and .parse(format=). The first defines the URL from which the coordinates of a molecular structure should be retrieved. The second defines the data format of the coordinates file (e.g., as “mmcif”, “pdb”, or “bcif”). For an exhaustive list of supported data formats, visit the documentation at https://github.com/molstar/molstar/blob/master/docs/file-formats.md.

### Troubleshooting

Table 2 lists issues that may arise with these protocols and their possible causes and solutions. Requests for further clarifications and error reports should be raised in the MolViewSpec code repository on GitHub (https://github.com/molstar/mol-view-spec/issues).

### Understanding Results

MolViewSpec produces files that define how a macromolecule should be visualized in 3D. For a comprehensive list of the supported visualization modes and features, refer to the documentation at https://github.com/molstar/molstar/blob/master/docs/extensions/mvs/README.md.

The output files are currently only supported by MolStar (https://molstar.org) (Sehnal et al., 2021), a widely used 3D molecular graphics viewer that can parse the specification and render molecular views accordingly. As a viewer-agnostic specification, the longer-term goal of MolViewSpec is to be supported by other popular molecular viewers such as PyMOL (https://pymol.org) and ChimeraX (https://www.cgl.ucsf.edu/chimerax/; Pettersen et al., 2021).

## Supplementary Material

Supinfo

## Figures and Tables

**Table 1:** Visualization Features Supported by MolViewSpec

Supported feature	Explanation
Download	Defines source of structural data
Parse	Parses structural data in various formats
Structure	Creates structure (including support for assemblies, crystal symmetry, multi-model structures)
Transform	Applies rotation and translation to a structure
Component	Selects substructures (components) of a structure
Representation	Defines standard structure representation modes
Color	Defines custom colors for selections
Label	Defines labels (textual representation in the 3D scene)
Tooltip	Defines tooltips (text shown when interacting with a component)
Camera	Sets camera position and orientation explicitly
Focus	Sets camera position and orientation automatically to focus a given component
Canvas	Sets background color
Annotations from URI	Creates component, coloring, labels, or tooltips based on MolViewSpec annotations from an external file referenced by URI
Annotations from source	Creates component, coloring, labels, or tooltips based on MolViewSpec annotations within the source structure file (mmCIF or BCIF)

**Table 2:** Troubleshooting

Problem	Possible cause	Solution
Script cannot be executed	Dependency on Python 3.9	Install MolViewSpec using Python interpreter 3.9 or higher
File parsing error	Trying to use unsupported coordinate file format	Use supported file formats such as mmCIF, BCIF, and PDB. Check that the format parameter matches actual file format.
	Gzipped data not supported	Use unzipped files.
Annotations fail to load	Wrong URI	Check the viewer tries to fetch the annotation file from the correct location. If using relative URI references, check that they resolve as intended (see Basic Protocol 2, step 8, for ensuring the availability of the annotation files).
	Server with annotations does not support cross-origin resource sharing (CORS)	Ensure the server sends the annotation files with CORS header; otherwise, the browser will block the request
	Wrong format, block header, category, or field name	Check that the parameters match actual file format and data
Viewer fails to load MolViewSpec state file	Newer format version	There are plans to extend the MolViewSpec functionality in the future. Check that the version number included in the MolViewSpec file is not higher than the viewer supports. Update the viewer if necessary.

## Data Availability

The codebase of MolViewSpec is public (https://github.com/molstar/mol-view-spec) and can be used to track issues or make contributions. The Python library is distributed using PyPI (https://pypi.org/project/molviewspec/). Mol* integration of MolViewSpec is available as Mol* extension (https://github.com/molstar/molstar/tree/master/src/extensions/mvs). Mol* is published using npm (https://www.npmjs.com/package/molstar).
